# Evaluation of Shade Integration of a Novel Universal-Shade Flowable Bulk-Filling Resin Composite

**DOI:** 10.3390/ma17235944

**Published:** 2024-12-04

**Authors:** Hirofumi Kaneko, Chiharu Kawamoto, Yu Toida, Ryotaro Yago, Di Wu, Yuan Yuan, Fei Chen, Monica Yamauti, Hidehiko Sano, Atsushi Tomokiyo

**Affiliations:** 1 Department of Restorative Dentistry, Graduate School of Dental Medicine, Hokkaido University, Kita 13 Nishi 7, Kita-ku, Sapporo 060-8586, Japan; k.cat.hiro5629@den.hokudai.ac.jp (H.K.); ryago@den.hokudai.ac.jp (R.Y.); 2 Department of Restorative Dentistry, Faculty of Dental Medicine, Hokkaido University, Kita 13 Nishi 7, Kita-ku, Sapporo 060-8586, Japan; wudi0526@den.hokudai.ac.jp (D.W.);; 3 Department of Stomatology, Beijing Tongren Hospital, Capital Medical University, No. 1, Dongjiaomingxiang Avenue, Dongcheng District, Beijing 100730, China; 4 Department of Biomedical and Applied Science, School of Dentistry, Indiana University, 1121 W. Michigan St., Indianapolis, IN 46202, USA

**Keywords:** CIEDE2000, color chart for image correction, colorimeter, SEM, simulated Class I cavity, structural color

## Abstract

Background: This study aimed to evaluate the color-matching and light transmission properties of a newly developed aesthetic flowable resin composite, OCFB-001. Methods: Rubber molds containing cylindrical cavities were filled with Estelite Sigma Quick, and 40 resin composite (CR) molds with simulated Class I cavities were prepared in shades A1, A2, A3, and A4, resulting in a total of 160 samples. Following bonding procedures, four different flowable resin composites (*n* = 10) were introduced into the cavities. The color difference (Δ*E*_00_) was calculated using two methods. A two-way analysis of variance was performed, and the interaction was significant, so a post hoc analysis was performed for each shade using Bonferroni’s correction. The morphology of the filler in each material was observed via scanning electron microscopy (SEM). Results: In the A1 shade, OCFB-001 demonstrated color differences comparable to those of other materials. However, in the A2, A3, and A4 shades, OCFB-001 exhibited significantly lower color differences (Δ*E*_00_) than the other materials, with a more consistent distribution. SEM analysis revealed that the OCFB-001 structure resembled that of Estelite Bulk Fill Flowable. Conclusions: OCFB-001 showed excellent shade matching in the A2, A3, and A4 ranges and good matching in the A1 shade, on par with existing universal-shade flowable bulk-fill resin composites.

## 1. Introduction

The advancement of adhesive dentistry, coupled with increasing aesthetic demands from patients, has established resin composites as the foremost restorative material in contemporary dental practice [1]. In dental restorative treatment, aesthetic requirements must be met by restoring the anatomy of the tooth and matching the color of the tooth and restorative material [2]. Natural teeth vary in transparency and shade between enamel and dentin and layering techniques have been used to achieve esthetic restorations [3,4,5]. However, layering techniques require the use of multiple types of resin composites of different shades. In addition, dental shade matching is most commonly performed subjectively by visual selection, which is dependent on the clinician’s experience, age, and physical condition [6]. These processes are complex and contribute to increased treatment times.

Universal-shade resin composites have recently emerged, attracting significant interest from clinicians due to their ability to match nearly all tooth shades, thereby simplifying restorative procedures [7].

The aesthetic success of these resin composites, particularly regarding the color tone compatibility with restored teeth, is intrinsically linked to their optical properties. These properties include light transmission, surface reflection, diffusion, absorption, and scattering [8,9]. The type and content of inorganic fillers and organic matrices further influence these optical properties, impacting the chromatic compatibility of the resin composites [10].

Conventional light-cured resin composites usually had to be placed at a thickness not exceeding 2 mm, due to the fact that the light intensity decreases below 2 mm from the surface layer, significantly reducing the depth of cure [11,12]. Therefore, in the case of restorative procedures for deep cavities, the resin composite had to be light-cured many times at a thickness of less than 2 mm, causing a prolonged treatment time. The introduction of bulk-fill resin composites, capable of filling cavities up to 4 mm in depth in a single increment, has significantly reduced treatment times compared to the traditional incremental filling technique [13].

In recent years, universal-shade resin composites have gained significant attention for their versatility in clinical applications [14]. These materials are engineered to match a wide range of tooth shades using a single shade, offering practical advantages in restorative dentistry. Notably, Omnichroma emerged as the first commercially available pigment-free universal-shade resin, utilizing structural coloration through 260 nm spherical fillers that enhance the reflection and refraction of specific light wavelengths [15]. Filler systems that combine spherical filler geometry with reduced average filler size are generally considered to have lower mechanical properties and increased susceptibility to age-related degradation, while filler systems incorporating spherical particles have been reported to lead to improved light transmission [16]. In addition, single-shade resin composites, such as Omnichroma, have an acceptable color-adjustment potential (CAP) [17,18]. This underlines how the concept of one-shade resin composites can be realized in a variety of ways and adds to the diversity found in the field of direct dental restorations [17,18].

Building on this foundational technology, a novel esthetic flowable resin composite, OCFB-001 (OCFB), has been developed.

Our previous research confirmed the excellent color adaptability of Omnichroma resin composite in a simulated 2 mm Class I cavity, supporting its effectiveness for universal-shade applications [19]. More recently, we have demonstrated that Omnichroma Flow also exhibits high color compatibility, characterized by relatively uniform reflectance and enhanced light transmission properties [20].

It has been reported that lightness decreases with the increasing cavity depth [21]. This phenomenon may be more pronounced in non-pigmented universal-shade resins. The esthetic performance of these materials may also be correlated with optical properties. In addition, most of the research reports on color differences of universal-shade resin are within 2 mm depth of cure, and there are few reports on color differences when bulk-fill type universal-shade resin is filled in deep cavities.

Thus, this study aims to assess the color compatibility and optical transmission characteristics of OCFB by fabricating a standardized 4 mm Class I cavity. The null hypothesis was that no significant differences in color compatibility and color parameters would be observed among the universal-shade flowable bulk-fill composite resins tested.

## 2. Materials and Methods

### 2.1. Specimen Preparation and Evaluation of Shade Matching

The details of the resin composites used in this study are shown in Table 1.

A 5 mm-thick, 10 mm-diameter acrylic block (Resin Disk Clear, Yamahachi Dental MFG., Co., Aichi, Japan) was used as the base material. The block was processed using a computer-aided design/computer-aided manufacturing (CAD/CAM) system (Ceramill mind, Amann Girrbach GmbH, Pforzheim, Germany) to create a cylindrical cavity with a diameter of 4 mm and a depth of 4 mm. The cutting process was performed using a CAD system in combination with a CAM machine (Ceramill motion2, Amann Girrbach GmbH, Pforzheim, Germany). A polymethyl methacrylate (PMMA) disc was utilized for this procedure, and three types of rotary cutting bars were employed: 0.6 mm diameter (Roto RFID 0.6 DMB DC, Amann Girrbach GmbH, Pforzheim, Germany), 1.0 mm diameter (Roto RFID 1.0 DMB DC, Amann Girrbach GmbH, Pforzheim, Germany), and 2.5 mm diameter (Roto RFID 2.5 DMB DC, Amann Girrbach GmbH, Pforzheim, Germany).

**Figure 1:**
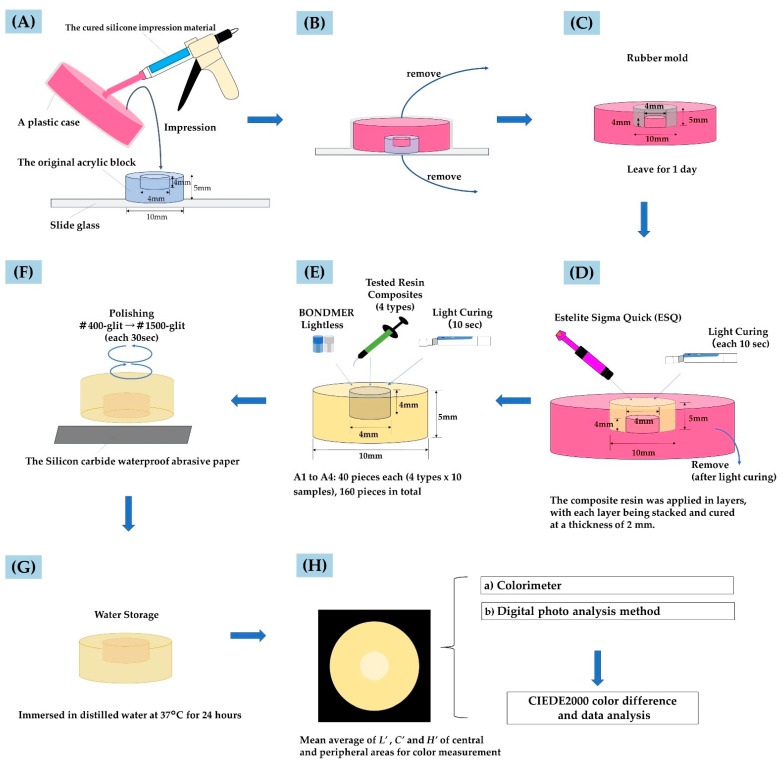
Diagrammatic representation of the specimen preparation for color measurement. The original acrylic block was placed on a glass slide with the cavity facing upward, filled with silicone impression material in a plastic case, and impressions were taken (**A**). A plastic case, the original acrylic block and slide glass were removed (**B**). The silicone mold was stored at room temperature in an incubator for 24 h to ensure complete reaction and to mitigate any potential hydrogen gas release (**C**). Using rubber molds, 160 frames were created in shades A1, A2, A3, and A4 (*n* = 40 per shades) by filling the molds with ESQ resin composite. The ESQ resin composite was light-cured with an LED curing unit (**D**). These cavities with ESQ framing were treated with a chemical-cure adhesive. Then, four types of resin composites were used to fill the simulated cavities (*n* = 10 per type).The four resin composites were filled in batches and light-cured for 10 s using an LED curing unit at an intensity of 1470 mW/cm^2^ (**E**). The surfaces of the restorations were polished with 400- and 1500-grit silicon carbide waterproof abrasive paper for 30 s (**F**). The polished specimens were stored in distilled water at 37 °C for 24 h (**G**). The Lab values were measured by two different methods and the color difference was calculated. Colorimetry was performed by two methods: (a) colorimetry using a colorimeter, and (b) an intraoral camera was used to capture images of specimens with color- and size-matching stickers placed on color reference tiles, which were then measured using image-processing software (digital photo analysis method) (**H**).

To replicate a simulated Class I cavity, silicone impression material (Sildefit wash type, Shofu Inc., Kyoto, Japan) was used to create a rubber mold from the acrylic block. The mold was designed to have a round cavity with a diameter of 10 mm and a height of 5 mm, as well as a central cylindrical projection matching the cavity dimensions (4 mm diameter and 4 mm depth). The impressions were made with the acrylic block positioned so that the cavity faced upward during the molding process (Figure 1A,B). The silicone mold was allowed to cure and was subsequently stored at room temperature in an incubator for 24 h to ensure complete reaction and to mitigate any potential hydrogen gas release during curing (Figure 1C). Using these molds, 160 frames were created in shades A1, A2, A3, and A4 (*n* = 40 per shades) by filling the molds with ESQ resin composite. The ESQ resin was light-cured with a light-emitted diode (LED) curing unit (PenCure 2000, Morita, Tokyo, Japan) at an intensity range of 660 to 760 mW/cm^2^ [19,20,22] (Figure 1D). As ESQ is not a bulk-fill-type resin, laminate filling was carried out at least twice with a thickness not exceeding 2 mm. The resin composition was slightly overfilled, and the surface was flattened under pressure with a transparent matrix and a glass slide and light-cured.

To simulate Class I cavities formed by the cylindrical projections, mock cavities (4 mm in diameter and 4 mm in height) were prepared for each shade, totaling 40 cavities per shade. These cavities were treated with a chemical-cure adhesive (Bondmer Lightless, Tokuyama Dental, Tokyo, Japan) according to the manufacturer’s instructions (Figure 1E).

Following surface treatment, four types of resin composites were used to fill the simulated cavities (*n* = 10 per type; Figure 1E). As with the ESQ framing, the resin composites were slightly overfilled and the surfaces were flattened by pressure with a transparent matrix and glass slides. The four resin composites were filled in batches and light-cured for 10 s using an LED curing unit (Elipar DeepCure-L, 3M ESPE, St. Paul, MN, USA) at an intensity of 1470 mW/cm^2^. After light curing, the surfaces of the restorations were polished with 400- and 1500-grit silicon carbide waterproof abrasive paper (Sankyo-Rikagaku Co., Ltd., Saitama, Japan) for 30 s (Figure 1F). The polished specimens were then stored in distilled water at 37 °C for 24 h (Figure 1G).

Colorimetry was performed by two methods: (i) colorimetry using a colorimeter, and (ii) an intraoral camera was used to capture images of specimens with color- and size-matching stickers (CASMATCH, BEAR Medic, Tokyo, Japan) placed on color reference tiles, which were then measured using image-processing software (digital photo analysis method).

(i) Colorimeter measurement

Lab values of the specimens were measured using a colorimeter (OFC-300, RC500, PaPaLaB Co., Tokyo, Japan) under a D65 light source (LDA9N-D-G, TOSHIBA, Tokyo, Japan) [14,15,19]. A color checker (ColorChecker, X-Rite INC., Tokyo, Japan) was used for calibration. The instrument was calibrated with a white background (L* = 93.2, a* = −0.3, b* = −1.6) and a black background (L* = 0.5, a* = 0.7, b* = −0.6) before each series of measurements (Figure 1H-a). Calibration was performed by first capturing the black background over the entire image display area, followed by capturing the white background over the entire image display area.

**Figure 2:**
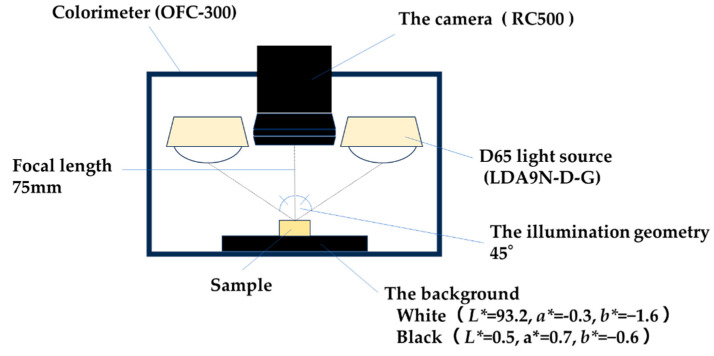
Photographic conditions (colorimeter).

**Figure 3:**
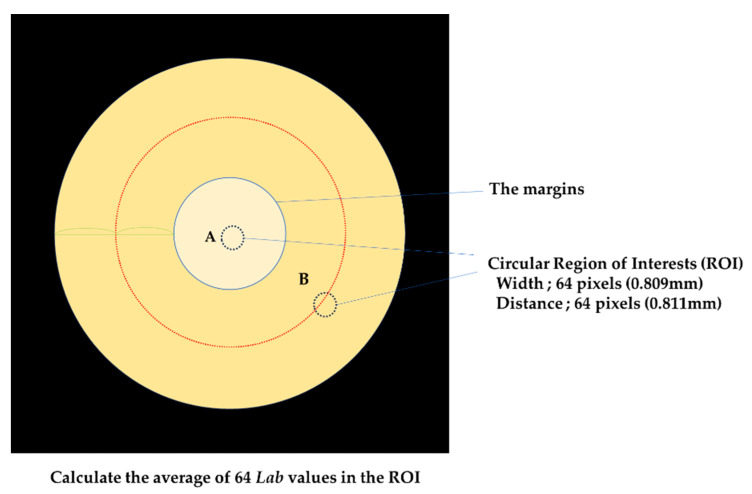
Measurement area dimensions (colorimeter). The measurement spot should be selected at least 0.3 mm away from the margin, avoiding the intersection between the four filled and framed resin composites. The four filled resin composites tested and the center of the sample were selected (A). The Frame resin composites, the midpoint between the margin and the edge were selected (B).

The illumination geometry employed for the reflectance measurements was bidirectional at 45°/0°. The following figures show the photographic conditions and the dimensional range of the measurement area for the colorimeter measurements (Figure 2 and Figure 3). The shooting distance was 75 mm.

To evaluate the color parameters, the measurement spot should be selected at least 0.3 mm away from the margin, avoiding the intersection between the four filled and framed resin composites [19,20,22]. Therefore, the center of the sample was selected for the filling area and the midpoint between the margin and the edge for the frame area. A circular region of image (ROI; width = 64 pixels (0.809 mm) and distance = 64 pixels (0.811 mm)) was used for colorimetry, and the average of Lab values at 64 locations in the region was calculated.

(ii) Digital photo analysis method

**Figure 4:**
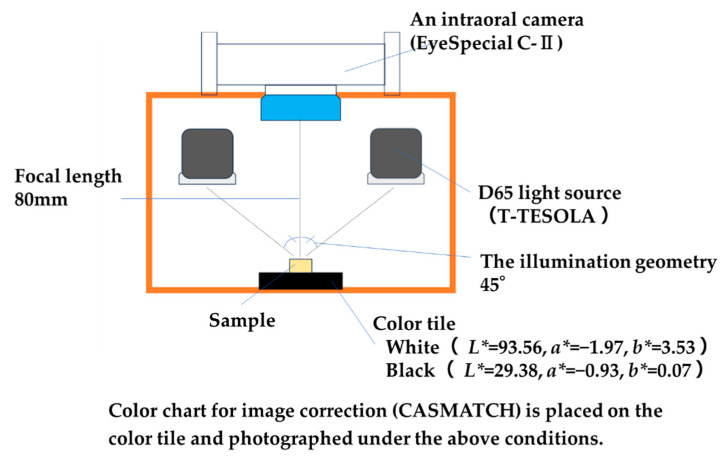
Photographic conditions (digital photo analysis method).

**Figure 5:**
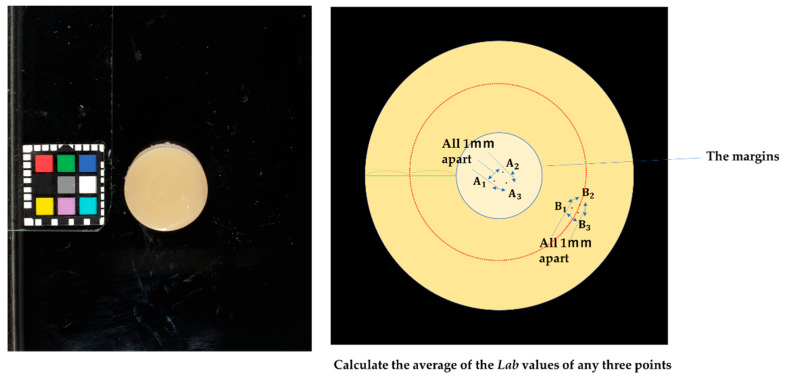
The typical image with color chart and measurement area dimensions (digital photo analysis method). The measurement spot should be selected at least 0.3 mm away from the margin, avoiding the intersection between the four filled and framed resin composites.

An intraoral camera (EyeSpecial C- II, Shofu Inc., Kyoto, Japan) was employed to obtain images of the specimens, along with color- and size-matching stickers (CASMATCH, BEAR Medic, Tokyo, Japan) placed on color reference tiles. The following figures show the photographic conditions and the dimensional range of the measurement area in the digital photo analysis method (Figure 4 and Figure 5). The images were captured under a D65 light source (T-TESOLA, TRUSCO, Tokyo, Japan), which approximates standard daylight conditions. The illumination geometry employed for the reflectance measurements was bidirectional at 45°/0°. Both a white tile (CM-101W, Konica Minolta, Tokyo, Japan) and a black tile (CM-101B, Konica Minolta) were utilized for reference. Photographs were taken using the camera’s low-glare mode to minimize surface reflections. The shooting distance was 80 mm.

Lab color values were extracted from the images using Adobe Photoshop (version 7.0, Adobe Inc., San Jose, CA, USA; Figure 1H-b). The color tiles used had Lab values of white background (L* = 93.56, a* = −1.97, b* = 3.53) and black background (L* = 29.38, a* = −0.93, b* = 0.07). Color correction of the images was performed in accordance with the guidelines provided in the CASMATCH correction manual to ensure accurate color representation. The measurement spot was selected at the center of the sample for the filling area and halfway between the margin and the edge for the frame area. Colorimetry was performed three times for both the center and the periphery of each sample, and the average value was calculated. The distance between each point was set to be 1 mm apart and recorded.

The CIE currently recommends using CIEDE2000, which utilizes the concepts of saturation and hue, and the color difference (Δ*E*_00_) is calculated using the following formula [23,24]. The Δ*E*_00_ was calculated using an Excel spreadsheet for calculating CIEDE2000 [25]:

(1) $$\Delta E_{00}={\left[{\left(\frac{\Delta L\prime }{K_{L}S_{L}}\right)}^{2}+{\left(\frac{\Delta C\prime }{K_{C}S_{C}}\right)}^{2}+{\left(\frac{\Delta H\prime }{K_{H}S_{H}}\right)}^{2}+R_{T}\left(\frac{\Delta C\prime }{K_{C}S_{C}}\right)\left(\frac{\Delta H\prime }{K_{H}S_{H}}\right)\right]}^{1/2}$$

where: $\Delta L^{\prime }=\left[L_{peripheral}-L_{Central}\right]$, $\Delta C^{\prime }=\left[C_{peripheral}-C_{central}\right],$ $\Delta H^{\prime }=\left[H_{peripheral}-H_{central}\right],$ *R_T_* is a rotation function that accounts for the interaction between chroma and hue differences, while the weighting functions *S_L_*, *S_C_*, and *S_H_* adjust the total color difference for variations in the location of the color difference. The parametric factors *K_L_*, *K_C_*, and *K_H_* are proposed to control the magnitude of tolerance judgments and adjust for scaling of acceptability rather than perceptibility. Previous research indicates that texture only affects lightness tolerances but not chroma or hue tolerances; therefore, a value of *K_L_* = 2 was proposed [20,24]. The perceptibility threshold (PT) and acceptability threshold (AT) are critical parameters for assessing the color matching of materials in esthetic dentistry [26,27]. Numerous studies have investigated the PT and AT for color differences (Δ*E_ab_* and Δ*E*_00_) within this field [28,29]. Based on these findings, the 50:50% PT in the CIELAB color space has been established at Δ*E_ab_* = 1.2, while the 50:50% AT has been reported as Δ*E_ab_* = 2.7 [30,31]. Corresponding values in the CIEDE2000 color difference formula (Δ*E*_00_) have been identified as 0.8 for PT and 1.8 for AT [30,31].

### 2.2. Measurement of Light Transmittance Characteristics

Five representative discs (diameter: 4 mm and thickness: 1 mm) from each tested filler-containing resin composite were prepared and immersed in distilled water at 37 °C for 24 h before testing. The two-dimensional distribution of transmitted light intensity for each disc (incidence angle: 0° and measurement range: −90° to +90°) was measured using a goniophotometer (Model GP-200, Murakami Color Research Laboratory, Tokyo, Japan) under controlled conditions (sensitivity: 950 and volume: 508) [32].

### 2.3. Filler Morphologies of Different Types of Filler Containing Resin Composites

The filler morphology of the four resin composites containing fillers, as well as the ESQ resin composite, was investigated using scanning electron microscopy (SEM; S-4000, Hitachi, Tokyo, Japan). Initially, the resin matrix was dissolved in acetone (Fujifilm Wako Pure Chemicals Industries Co., Ltd., Osaka, Japan). The test specimens were subsequently rinsed with water and allowed to air dry for 5 s. Following additional drying, the specimens were mounted on aluminum stubs and sputter-coated with platinum-palladium (Pt-Pd) ions (E-1030, Hitachi, Tokyo, Japan). Observations were conducted using SEM at an acceleration voltage of 10 kV. The filler morphology was examined at magnifications of ×10,000 and ×20,000.

### 2.4. Comparison of Two Colorimetric Methods

Instrumental measurements are generally considered to be unreliable between devices [33]. The correlation coefficients were analyzed to evaluate the relationship between the color differences determined by the two measurement approaches: the colorimetric method and the digital photo analysis method. To compare the accuracy of colorimetric measurements (Δ*E*_00_) obtained by colorimeter and digital photo analysis methods, statistical analysis was performed using statistical analysis software (SPSS 28.0, IBM, Armonk, NY, USA). The Mann–Whitney U test was performed using the color difference (Δ*E*_00_) in the same material and the same shade. The significance level was set at 5%.

### 2.5. Statistical Analysis

Statistical analysis was performed using statistical software (SPSS 28.0, IBM, Armonk, NY, USA). The significance level was set at 5%. Two-way ANOVA was used to examine the effects of the four materials and four shades on the color difference (Δ*E*_00_). If the statistical treatment showed an interaction between the material and shade, a back-test using the Bonferroni method was performed to examine the main effect for each shade.

## 3. Results

### 3.1. Evaluation of Shade Matching

Colorimeter results for each material are shown below (Table 2 and Table 3). On the white background, *L*′ showed smaller values from A1 to A4, even though the same composite resin was used. *C*′ showed larger values from A1 to A4, *H*′ showed little variation but a slight decreasing trend from A1 to A4, and *L*′ showed a smaller value from A1 to A4, even though the same composite resin was used. On the black background, *L*′ showed small values from A1 to A4, as on the white background, but the values were smaller than on the white background. *C*′ showed an increasing trend, while *H*′ showed a decreasing trend. On both white and black backgrounds, *L*′ showed higher values for EsBF, while *C*′ and *H*′ showed higher values for OCBF in almost all cases.

In addition, *L*′, *C*′, *H*′, and color difference (Δ*E*_00_) obtained by the digital photo analysis method are shown in Table 4 and Table 5.

### 3.2. Color Difference

**Figure 6:**
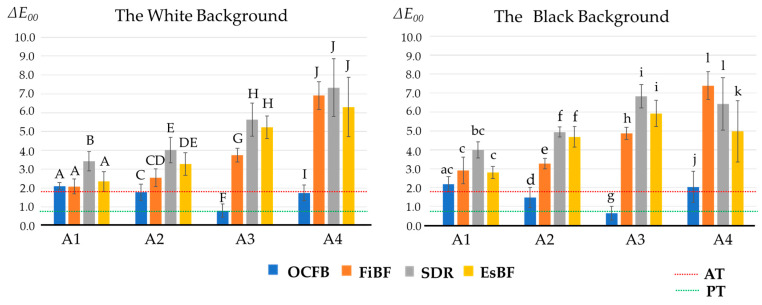
The color difference between four tested materials and resin composites (colorimeter). The perception threshold (PT) in CIEDE2000 was set at 0.8, and the acceptance threshold (AT) at 1.8. The letter differences indicate significant differences between materials of the same shade (*p* < 0.05).

**Figure 7:**
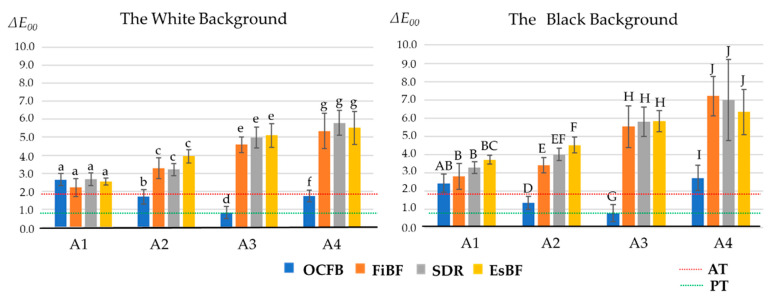
The color difference between the four tested materials and resin composites (digital photo analysis method). The perception threshold (PT) in CIEDE2000 was set at 0.8, and the acceptance threshold (AT) at 1.8. The letter differences indicate significant differences between materials of the same shade (*p* < 0.05).

Furthermore, the color difference (Δ*E*_00_) is shown in the graphs in Figure 6 and Figure 7. The color difference (Δ*E*_00_) between each material and the ESQ mold is shown below (Figure 6). The color difference (Δ*E*_00_) between OCFB and the ESQ mold was 2.10 ± 0.2 (A1), 1.78 ± 0.2 (A2), 0.81 ± 0.4 (A3), and 1.74 ± 0.4 (A4) for the white background, and 2.17 ± 0.4 (A1), 1.48 ± 0.5 (A2), 0.64 ± 0.4 (A3), and 2.03 ± 0.8 (A4) for the black background. The Δ*E*_00_ of OCFB, FiBF, SDR, and EsBF were similar for A1, but the Δ*E*_00_ of FiBF, SDR, and EsBF became larger as the shade became darker. On the other hand, the Δ*E*_00_ of OCFB was almost the same as or lower than that of A1, even as the shade became darker. Since the statistical treatment showed an interaction between the material and shade, a Bonferroni back-test for each shade showed that OCFB was significantly lower than FiBF, SDR, and EsBF for A2, A3, and A4 on white and black backgrounds (*p* < 0.05; Figure 6).

The Δ*E*_00_ of OCFB, FiBF, SDR, and EsBF were similar in the A1 shade, but as the shade became darker, the Δ*E*_00_ of FiBF, SDR, and EsBF became larger. On the other hand, the Δ*E*_00_ of OCFB remained almost the same as or lower than that of the A1 shade, even as the shade became darker. Statistical analysis showed an interaction effect between material and shade. When back-testing was performed for each shade using the Bonferroni method, OCFB was significantly lower than FiBF, SDR, and EsBF for shades A2, A3, and A4 on white and black backgrounds (*p* < 0.05; Figure 7).

**Figure 8:**
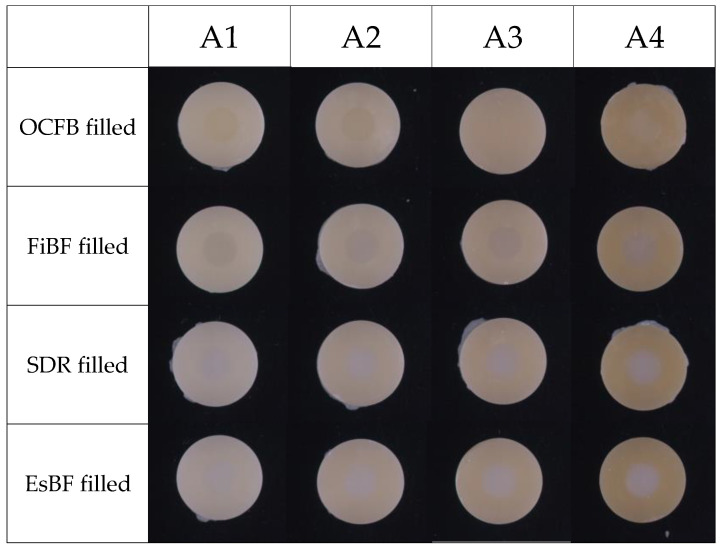
The images of the filled tested resin composites.

From the colorimeters, a representative photograph of a sample after each filling is shown below (Figure 8). In the A1 and A2 groups, the filled resin composites generally exhibited a close shade match with the resin mold. Conversely, in the FiBF, SDR, and EsBF groups, an increase in the whiteness of the filled areas became more evident as the shade darkened. In the OCFB group, the filled area demonstrated effective color blending with the surrounding resin, particularly in the A3 and A4 shades. Notably, OCFB consistently achieved superior blending with the surrounding color in these darker shades.

### 3.3. Measurement of Light Transmittance Characteristics

**Figure 9:**
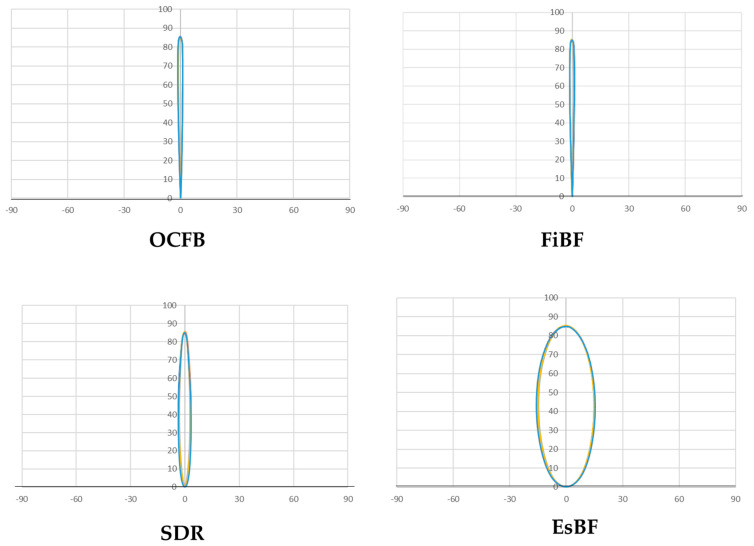
Two-dimensional analysis of light transmittance properties. The horizontal axis of the graph represents the measurement range (°), and the vertical axis represents the intensity (relative value, no unit). The light transmittance of each material was measured five times, with each measurement represented by a distinct line in the graph, differentiated by five colors.

The two-dimensional distribution graph of the light transmittance properties of the resin composites tested (Figure 9) revealed that the EsBF-filled group had a wider distribution of light transmission intensity than the other filling groups. This indicates that the diffusion behavior was enhanced compared to the other filling groups. The SDR-filled group also showed a wider distribution of light transmission intensity than the OCFB- and FiBF-filled groups. The horizontal axis of the graph represents the measurement range (°), and the vertical axis represents the intensity (relative value, no unit).

### 3.4. Filler Morphologies of Different Types of Filler Containing Resin Composites

**Figure 10:**
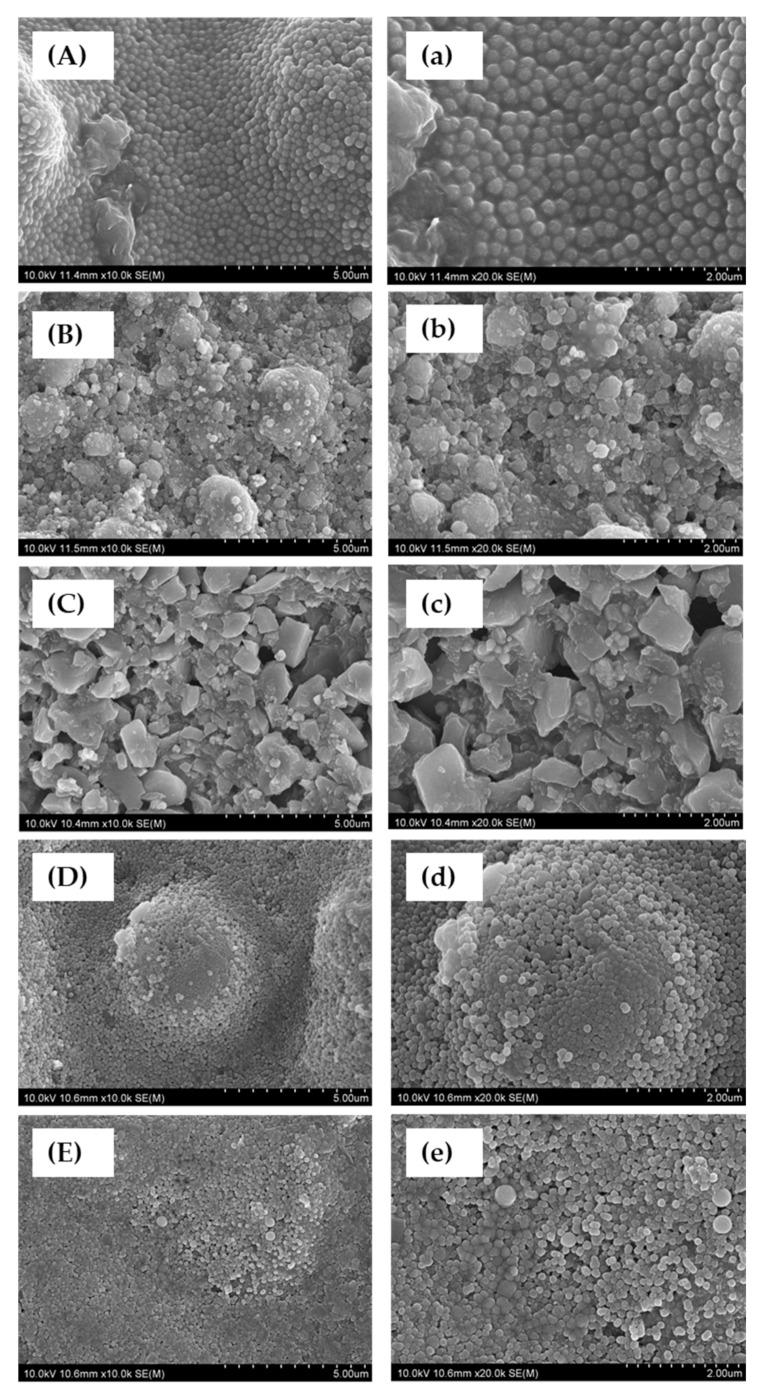
SEM images display the fillers within the tested resin composites and the resin composite frame. The left column presents images at ×10,000 magnification, while the right column showcases images at ×20,000 magnification. (**A**,**a**) OCFB fillers, (**B**,**b**) FiBF fillers, (**C**,**c**) SDR fillers, (**D**,**d**) EsBF fillers, and (**E**,**e**) the ESQ resin composite frame.

The SEM images were captured at ×10,000 and ×20,000 magnifications, revealing distinct variations in the morphology, dimensions, and distribution of the filler particles within different resin composites and the resin composite framework (Figure 10).

OCFB showed a regular distribution of spherical nanofiller particles (Figure 10A,a). FiBF showed aggregates of nanoclusters and nanofillers (Figure 10B,b). SDR showed an irregular distribution of polygonal particles (Figure 10C,c). EsBF showed an image similar to OCFB (Figure 10D,d). ESQ showed spherical nanofiller particles, and occasionally, large irregular spherical particles were observed (Figure 10E,e).

### 3.5. Comparison of the Two Colorimetric Methods

The correlation coefficients calculated to compare the two methods revealed varying levels of agreement across different conditions. On the white background, the coefficients were as follows: 0.81 for OCFB, 0.80 for FiBF, 0.73 for SDR, and 0.78 for EsBF. These values, ranging from 0.7 to 0.9, indicate a generally strong positive correlation among the tested methods. On the black background, the coefficients were 0.77 for OCFB, 0.85 for FiBF, 0.73 for SDR, and 0.59 for EsBF. While most methods maintained a strong correlation under this condition, EsBF demonstrated a weaker relationship. Overall, the findings suggest that the correlations on the white background were consistently strong, whereas the black background showed greater variability, particularly for EsBF (Table 6).

**Figure 11:**
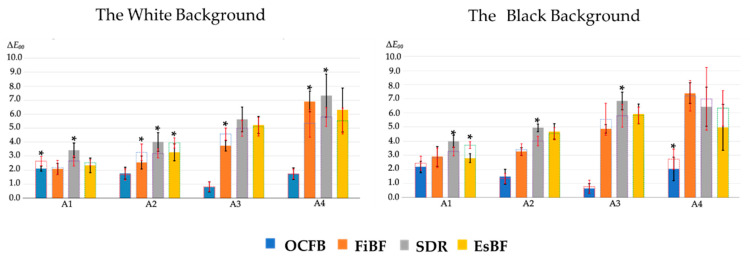
Comparison of colorimeters and CASMATCH (the white and black backgrounds). The solid line in the graph shows the color difference of the colorimeter, and the dashed line shows the color difference of the digital photo analysis method. The black color in the error bar indicates the colorimeter and the red color indicates the digital photo analysis method. * In the figure indicates a significant difference between the two groups (*p* < 0.05).

On a white background, no significant differences were observed between the two measurement methods among the nine groups (*p* > 0.05; Figure 11): OCFB (A2, A3, and A4), FiBF (A1), SDR (A3), and EsBF (A1, A3, and A4). However, significant differences were found in the remaining 7 groups (*p* < 0.05). On a black background, no significant differences were found in 11 groups: OCFB (A1, A2, and A3), FiBF (A1, A2, A3, and A4), SDR (A4), and EsBF (A2, A3, and A4; *p* > 0.05; Figure 11). However, significant differences were found in the remaining 5 groups (*p* < 0.05).

## 4. Discussion

In this study, color differences were assessed using two methods: a colorimeter and a digital measurement technique. A comparative analysis revealed a correlation between these two approaches. To enhance both intra- and inter-rater reliability in visual and instrumental tooth color matching, as well as to achieve improved esthetic outcomes, the integration of color-matching instruments as supplementary tools in routine dental practice is recommended [34].

However, specialized instruments, such as colorimeters, are often cost-prohibitive and are not routinely available in most dental clinics [35]. The CASMATCH system, utilizing computerized image-correcting color charts, offers an alternative approach by standardizing the tonality and dimensions of photographic images for the subject. Similarly, Photoshop has demonstrated high validity and reliability for color selection purposes [36].

Given the widespread availability of digital cameras in dental practices [37] and the relatively low cost of CASMATCH compared to colorimeters, the use of CASMATCH in combination with an intraoral camera presents a more accessible and practical alternative for color measurement. This approach may facilitate broader adoption and ease of implementation in clinical settings. In recent years, universal-shade resin composites capable of accommodating a wide range of color shades within a single shade have been introduced to the market. This study focused on evaluating the color compatibility of bulk-fill-type resin composites, which allow for efficient, single-step cavity filling.

Our findings demonstrated that the colorimetric parameters *L*′, *C*′, and *H*′ of the tested resin composites were significantly influenced by the shade of the resin composite mold, particularly as the mold shade darkened. Specifically, *L*′ decreased, *C*′ increased, and *H*′ decreased as the mold shade progressed from A1 to A4. These results align with previous research reporting a decrease in *L*′ values as the cavity depth increased in a simulated fossa cavity of 2 mm in depth. In this study, a deeper simulated first-class cavity of 4 mm was utilized, confirming a consistent trend of diminished lightness with increasing cavity depth [21].

The observed changes in color parameters can be attributed to intrinsic properties of the resin composites, such as light scattering, absorption, reflectance, and translucency. When light interacts with a resin composite, it encounters the resin matrix, filler particles, pigments, and background color, which collectively influence the perceived color [38]. Notably, the interplay of light scattering and absorption modifies the background color through a filtering effect governed by the translucency of the material [23]. Furthermore, the thickness of the resin composite and the reflectance of the underlying background were critical in determining the resultant color coordinates [23]. These principles also apply to universal-shade resins, as demonstrated in our study.

Additionally, scanning electron microscopy (SEM) analysis revealed variations in filler morphology among the examined materials. For instance, OCFB and EsBF exhibited fillers with similar shapes but differing sizes. The refractive index of the filler and matrix, as well as the light diffusivity of the resin composite, are influenced by the filler content and matrix composition. The optical diffusivity of EsBF was notably higher than that of the other tested materials, including OCFB. This difference may be attributed to variations in the filler particle size and distribution.

Previous research has indicated that resin composites with smaller, irregularly shaped fillers generally exhibit higher light transmittance and sharper angular distribution peaks compared to those with larger, spherical fillers. Interestingly, while SDR contains irregular fillers, its diffusivity was surpassed by EsBF in this study. This discrepancy is likely due to the particle size of the fillers in EsBF, emphasizing the importance of filler morphology in determining optical properties.

In conclusion, the interaction of material properties, such as filler shape, size, and distribution, with optical characteristics underscores the complexity of color compatibility in resin composites. These findings provide valuable insights into the factors influencing the performance of universal-shade and bulk-fill resins in restorative dentistry.

While the CIELAB formula (Δ*E_ab_*) is frequently applied in dental procedures due to its established reliability in quantifying color, the CIEDE2000 formula (Δ*E*_00_) offers a more advanced approach [23,26]. The latter provides a closer correlation with perceptual color differences, as judged by human vision [23,25,39]. Therefore, the CIEDE2000 formula was selected for use in this study to assess color differences, considering its demonstrated advantages in aligning with human visual perception.

As mentioned earlier, corresponding values in the CIEDE2000 color difference formula (Δ*E*_00_) have been identified as 0.8 for PT and 1.8 for AT [30,31]. The color difference measurements of OCFB, as evaluated using a colorimeter, demonstrated satisfactory color matching, with Δ*E*_00_ values below the acceptable threshold (AT) for shades A2 and A4 against a white background and for shade A2 against a black background. Additionally, OCFB exhibited the best color matching, with Δ*E*_00_ values below the perceptibility threshold (PT) for shade A3 on both white and black backgrounds. Similarly, the CASMATCH analysis indicated that OCFB achieved good color differences below the AT for shades A2, A3, and A4 on the white background, as well as for shade A2 on the black background. Notably, the best color difference, below the PT, was observed for shade A3 on the black background.

In the A1 shade measured with a colorimeter, the Δ*E*_00_ values of OCFB, FiBF, and EsBF were nearly identical when measured against the black background, while SDR exhibited significantly higher values (*p* < 0.05). On the white background, OCFB showed significantly lower color differences compared to SDR (*p* < 0.05).

Omnichroma Flow contains uniform spherical ultra-nanofiller particles with an average diameter of 260 nanometers and, furthermore, contains no pigment and has high transparency [40].

Similar to Omnichroma and Omnichroma Flow, OCFB also contains uniform spherical ultra-nanofiller particles with an average diameter of 260 nanometers and contains no pigments. Furthermore, the bulk-fill-type resin composite allows filling of 4 mm cavities in a batch.

The structural color depends on the distribution of refractive indices, and the difference between the matrix and filler refractive indices must be considered, with the refractive index of the filler particles ranging from 1.47 to 1.52, corresponding to the refractive index of the resin matrix [10]. Arai et al. also found that the appearance of the structural color depends not only on the size of the filler but also on the filler content [41]: the filler content of Omnichroma Flow is 70% and the matrix resin is UDMA [40]. In our previous study, Omnichroma Flow showed a better color match compared to other esthetic resin composites at a cavity depth of 2 mm [20]. This was due to the structural coloration. On the other hand, the filler content of OCFB was 69%, which is almost the same as that of Omnichroma Flow, and the matrix resins are UDMA and TEGDMA. This indicates that OCFB also has a characteristic structural color, which may serve as an explanation for the better esthetic results observed in this study. In other words, OCFB contains spherical ultra-nanofiller particles with an average diameter of 260 nm, which contribute to red–yellow wavelength reflection and contribute to shade matching.

A previous study showed that shade matching of resin composites is facilitated by the light transmittance and light diffusivity of the material [42].

The color of a resin composite is affected by the light scattering, absorption properties, reflectance, and translucency of the material [43]. When light enters a resin composite, it interacts with the resin matrix, fillers, pigments, and background color and is simultaneously perceived as a specific color [38]. The interaction of light scattering and absorption changes the color parameters due to the filtering effect caused by the translucency of the material, affecting the background color [34]. Both the thickness of the resin composite and the reflectance of the background play an important role in determining the color coordinates [23].

A previous study reported a statistically significant difference (*p* < 0.05) in translucency between resin composites with and without pigments, with translucency decreasing as the pigment concentration increased [44]. There is also a negative correlation between material thickness and transparency, with transparency decreasing with increasing thickness [44]. This is because as material thickness increases, light travels a longer path through the sample and encounters more interfaces between particles and the matrix, resulting in increased scattering at these interfaces [45].

Nevertheless, in bulk-filled resin composites, a thickness of 4 mm can be polymerized directly according to the manufacturer. This may be explained by its satisfactory translucency (OCFB, FiBF, and SDR) [45]. The compatibility of the refractive index of the resin matrix and filler particles is important to achieve high translucency in resin composites [46]. Therefore, future studies will need to evaluate the light transmittance and spectral reflectance of each bulk-filled resin composite using a spectrophotometer.

In our previous study, the flowable type of esthetic resin composite, Omnichroma Flow, showed superior color matching compared to other esthetic resin composites at a cavity depth of 2 mm [20]. On the other hand, a cavity depth of 4 mm was used in this study. The results showed that the bulk-filled resin composite OCFB-001 exhibited perceptible color matching up to the A1–A4 shades at a cavity depth of 4 mm, while other flowable resin composites exhibited perceptible color matching at the A1 shade, but for the surrounding cavity shades A2, A3, and A4, the color differences were discernible.

This may be because OCFB does not contain pigments and exhibits excellent color matching due to its coloration by structural color, while the other three materials have adjusted the amounts of pigments and the refractive index of the matrix and filler to match a white shade, such as A1.

Depending on its structural color, OCFB reflects yellow wavelengths in light-color cavities, such as the A1 shade, and red wavelengths in dark-color cavities, such as the A4 shade, in harmony with the cavity wall color.

The other three materials, on the other hand, would have had perceptible color matching in the lighter materials, such as the A1 shade, but the pigments and low lightness would have made harmonization more difficult and the color differences more pronounced.

Nevertheless, this study examined the color matching of only universal shades and did not compare the results with the results of actual dentists’ rigorous shade matching and filling. For the three commercial materials used, it is possible that they may have shades that may better match certain colors. Conventional composite resin restorations for a cavity depth of 4 mm are more difficult to restore because light penetration into the cavity floor is weak and prone to under-polymerization. In addition, deeper cavities are usually darker in color than the cavity floor, and the use of the cavity floor for light reflection to improve esthetics also has an effect, making color matching difficult [46]. As mentioned earlier, OCFB was shown to have good tonal conformity in a 4 mm simulated cavity. From this, it can be inferred that satisfactory translucency was achieved. In this study, a 4 mm monochromatic simulated Class I cavity was used, but this is not the case in Class II–IV cavities, which do not have a dentin backing. Further study is needed because in natural teeth, the color varies among the superficial enamel, the dentin behind it, and the dentin adjacent to the pulp.

Based on the above, the null hypothesis that “there is no significant difference in color match and color parameters among the tested universal-shade flowable bulk-fill composite resins in the 4 mm standard cavity” was rejected.

The application of filler forms for shade matching represents a recent advancement in dental material science. This technique relies on the light absorption and emission properties of resin composites to achieve precise shade matching. Notably, super nano-filled resin composites, which derive their shading primarily from the structural color, present a potential advantage due to the alignment of their filler particles with visible-light wavelengths. This alignment minimizes photochemical degradation and reduces color distortion over time, thereby enhancing shade stability [20]. Consequently, the incorporation of optically controlled filler-based systems (OCFBs) may streamline treatment by reducing the time required for shade-matching and layering techniques. Furthermore, this approach may lower the technical sensitivity of the process, enabling practitioners of varying skill levels to achieve optimal aesthetic outcomes with greater ease. However, as this investigation was conducted under in vitro conditions, further research in clinical settings is imperative to validate these findings and assess their practical implications.

## 5. Conclusions

OCFB-001 demonstrated excellent color compatibility within the shade range of A2 to A4, and comparable color compatibility to existing universal bulk-fill resin composites in the A1 shade. Additionally, the use of OCFB-001 is expected to streamline procedures and enhance esthetic outcomes. These findings underscore its clinical utility and suggest potential future research directions, such as validation under natural lighting or using natural teeth as testing conditions.

## Figures and Tables

**Table 1:** Resin composites, their manufacturers, organic matrices, presence of pigments, and Lot numbers.

	Resin Composites (Code) (Manufactures)	Organic Matrices	Pigments	Lot Numbers
Filling Resin Composites	OCFB-001 (OCFB) (Tokuyama Dental, Tokyo, Japan)	UDMA	-	21l02
		TEGDMA		
	Filtek Bulk Fill Flowable (FiBF) (3M ESPE, St. Paul, MN, USA)	Bis-GMA	+	NF34174
		UDMA		
		Bis-EMA (6)		
	SDR flow+ (SDR) (Dentsply Sirona, Charlotte, NC, USA)	TEGDMA	+	2205000974
		EBPADMA		
	Estelite Bulk Fill Flowable (EsBF) (Tokuyama Dental, Tokyo, Japan)	Bis-GMA	+	8228
		Bis-MPEPP		
		TEGDMA		
Mold	Estelite Sigma Quick (ESQ) (Tokuyama Dental, Tokyo, Japan)	Bis-GMA	+	175082
		TEGDMA		

UDMA, urethane dimethacrylate; TEGDMA, triethylene glycol dimethacrylate; Bis-GMA, bisphenol A-glycidyl methacrylate; Bis-EMA (6), bisphenol A-ethoxylated dimethacrylate; EBPADMA, ethoxylated bisphenol A-dimethacrylate; Bis-MPEPP, 2,2′-bis(4-methacryloxyate polyethoxyphenyl)propane.

**Table 2:** *L*′, *C*′, *H*′, and Δ*E*_00_ obtained from measurements with the colorimeter (white background).

ESQ’s Shade	Code	*L*′	*C*′	*H*′	Δ*E*_00_
A1	OCFB	80.2(0.6)	25.5(0.6)	254.0(1.4)	2.10(0.2)
	FiBF	81.0(0.5)	23.6(0.4)	250.7(1.3)	2.09(0.4)
	SDR	81.6(0.8)	20.9(0.6)	247.2(1.9)	3.43(0.5)
	EsBF	82.0(0.4)	22.1(0.4)	250.5(1.5)	2.35(0.5)
A2	OCFB	79.6(0.3)	28.5(0.3)	250.2(1.0)	1.78(0.2)
	FiBF	79.5(0.5)	26.4(0.2)	247.0(1.0)	2.55(0.4)
	SDR	79.6(0.7)	24.0(0.6)	243.8(1.2)	4.02(0.5)
	EsBF	80.5(0.3)	24.5(0.5)	245.0(0.9)	3.28(0.5)
A3	OCFB	76.7(0.4)	30.0(0.3)	248.9(0.7)	0.81(0.4)
	FiBF	76.4(0.6)	28.0(0.5)	245.6(0.9)	3.74(0.4)
	SDR	77.3(0.6)	25.2(0.6)	241.9(1.6)	5.64(0.9)
	EsBF	78.1(0.4)	25.3(0.3)	243.2(1.0)	5.22(0.6)
A4	OCFB	71.6(0.4)	31.4(0.6)	248.6(0.9)	1.74(0.4)
	FiBF	71.0(0.5)	29.4(0.2)	245.1(0.8)	6.90(0.7)
	SDR	72.1(0.6)	27.2(0.6)	242.6(0.9)	7.33(1.5)
	EsBF	72.4(0.5)	26.6(0.6)	241.5(1.1)	6.30(1.6)

**Table 3:** *L*′, *C*′, *H*′, and Δ*E*_00_ obtained from measurements with the colorimeter (black background).

ESQ’s Shade	Code	*L*′	*C*′	*H*′	Δ*E*_00_
A1	OCFB	72.7(0.9)	21.6(0.6)	259.7(2.2)	2.17(0.4)
	FiBF	72.4(0.6)	18.2(0.4)	256.6(2.0)	2.91(0.7)
	SDR	69.6(0.6)	12.4(0.3)	265.5(2.7)	3.99(0.4)
	EsBF	74.0(0.5)	14.2(0.5)	268.2(5.9)	2.79(0.3)
A2	OCFB	68.0(0.5)	20.6(0.3)	260.1(1.6)	1.48(0.5)
	FiBF	66.7(0.4)	17.5(0.1)	258.5(2.4)	3.27(0.3)
	SDR	68.0(0.6)	15.1(0.4)	256.6(3.1)	4.94(0.3)
	EsBF	72.4(0.7)	16.3(0.6)	258.1(3.7)	4.69(0.5)
A3	OCFB	65.9(0.4)	22.6(0.4)	256.7(0.9)	0.64(0.4)
	FiBF	64.7(0.2)	19.6(0.5)	252.0(1.7)	4.87(0.3)
	SDR	66.0(0.5)	16.9(0.4)	250.9(1.6)	6.83(0.6)
	EsBF	70.1(0.8)	17.8(0.8)	252.1(1.8)	5.92(0.7)
A4	OCFB	61.6(0.7)	24.9(0.5)	255.7(0.8)	2.03(0.8)
	FiBF	59.9(0.8)	22.0(0.4)	249.5(0.9)	7.39(0.7)
	SDR	61.4(0.5)	20.0(0.4)	246.3(3.7)	6.43(1.4)
	EsBF	65.2(0.6)	20.1(0.6)	246.9(4.0)	4.97(1.6)

**Table 4:** *L*′, *C*′, *H*′, and Δ*E*_00_ obtained from measurements with the digital photo analysis method (white background).

ESQ’s Shade	Code	*L*′	*C*′	*H*′	Δ*E*_00_
A1	OCFB	86.6(1.8)	28.6(1.5)	267.2(0.7)	2.65(0.3)
	FiBF	86.9(1.1)	27.3(0.5)	265.3(0.6)	2.20(0.5)
	SDR	87.0(1.0)	24.4(0.6)	265.9(0.3)	2.66(0.4)
	EsBF	86.2(1.6)	24.4(1.0)	265.4(1.4)	2.53(0.21)
A2	OCFB	86.4(1.2)	33.7(0.4)	264.2(0.7)	1.69(0.4)
	FiBF	86.5(0.7)	30.8(0.4)	262.1(0.7)	3.27(0.6)
	SDR	86.9(0.6)	28.8(0.4)	261.9(0.7)	3.20(0.3)
	EsBF	86.6(1.5)	27.8(0.6)	261.5(0.6)	3.94(0.4)
A3	OCFB	83.5(1.2)	35.9(0.4)	261.6(0.6)	0.81(0.3)
	FiBF	83.2(1.7)	32.6(0.5)	258.4(0.7)	4.58(0.4)
	SDR	84.6(0.9)	30.1(0.8)	258.8(0.5)	4.99(0.6)
	EsBF	85.1(0.6)	29.6(0.4)	258.3(0.5)	5.10(0.7)
A4	OCFB	77.2(0.7)	38.4(0.7)	257.0(0.5)	1.73(0.3)
	FiBF	75.7(2.1)	35.1(0.5)	254.2(1.2)	5.35(1.0)
	SDR	77.9(0.7)	32.9(0.6)	254.7(0.5)	5.80(0.7)
	EsBF	77.4(1.5)	31.0(0.7)	253.3(1.1)	5.51(0.9)

**Table 5:** *L*′, *C*′, *H*′, and Δ*E*_00_ obtained from measurements with the digital photo analysis method (black background).

ESQ’s Shade	Code	*L*′	*C*′	*H*′	Δ*E*_00_
A1	OCFB	83.9(0.7)	25.1(0.5)	269.4(0.2)	2.43(0.5)
	FiBF	82.5(1.1)	21.7(0.9)	268.5(0.7)	2.81(0.7)
	SDR	83.8(1.1)	19.5(0.7)	269.2(0.5)	3.27(0.3)
	EsBF	83.8(1.7)	19.7(0.5)	269.4(0.9)	3.72(0.3)
A2	OCFB	81.5(1.1)	28.4(0.9)	266.8(0.7)	1.36(0.4)
	FiBF	81.6(0.9)	24.7(0.7)	266.3(0.7)	3.41(0.4)
	SDR	82.1(1.0)	23.1(0.6)	266.1(0.8)	4.01(0.3)
	EsBF	82.0(2.2)	22.7(0.3)	265.1(1.3)	4.52(0.4)
A3	OCFB	78.5(1.3)	31.1(0.5)	264.2(1.3)	0.78(0.5)
	FiBF	78.2(1.1)	27.8(0.6)	261.2(1.0)	5.54(1.1)
	SDR	79.7(0.7)	24.8(0.5)	262.1(1.3)	5.80(0.8)
	EsBF	79.2(1.6)	24.3(0.5)	261.3(0.7)	5.82(0.6)
A4	OCFB	73.2(1.3)	34.2(0.7)	259.4(1.1)	2.72(0.7)
	FiBF	71.3(1.3)	30.0(0.4)	255.0(1.0)	7.21(1.1)
	SDR	73.0(1.9)	27.5(0.7)	255.9(1.0)	6.99(2.2)
	EsBF	73.5(1.0)	26.8(0.6)	255.0(1.3)	6.33(1.3)

**Table 6:** Correlation coefficients of the color differences between colorimeters and digital photo analysis measurement methods.

	OCFB	FiBF	SDR	EsBF
The White Background	0.81	0.80	0.73	0.78
The Black Background	0.77	0.85	0.73	0.59

The values from 0 to less than 0.3 indicate negligible correlation, 0.3 to less than 0.5 indicate a very weak correlation, 0.5 to less than 0.7 indicate a moderate correlation, 0.7 to less than 0.9 indicate a strong correlation, and 0.9 or greater indicate a very strong correlation.

## Data Availability

The original contributions presented in the study are included in the article, further inquiries can be directed to the corresponding author.

## References

[1] Sidhu S.K., Ikeda T., Omata Y., Fujita M., Sano H. (2006). Change of color and translucency by light curing in resin composites. Oper. Dent..

[2] Lucchese A., Storti E. (2011). Morphological characteristics of primary enamel surfaces versus permanent enamel surfaces: SEM digital analysis. Eur. J. Paediatr. Dent..

[3] Ikeda T., Sidhu S.K., Omata Y., Fujita M., Sano H. (2005). Colour and translucency of opaque-shades and body-shades of resin composites. Eur. J. Oral Sci..

[4] Miotti L.L., Santos I.S., Nicoloso G.F., Pozzobon R.T., Susin A.H., Durand L.B. (2017). The Use of Resin Composite Layering Technique to Mask Discolored Background: A CIELAB/CIEDE2000 Analysis. Oper. Dent..

[5] Perez B.G., Gaidarji B., Palm B.G., Ruiz-López J., Pérez M.M., Durand L.B. (2022). Masking ability of resin composites: Effect of the layering strategy and substrate color. J. Esthet. Restor. Dent..

[6] Hardan L., Bourgi R., Cuevas-Suárez C.E., Lukomska-Szymanska M., Monjarás-Ávila A.J., Zarow M., Jakubowicz N., Jorquera G., Ashi T., Mancino D. (2022). Novel Trends in Dental Color Match Using Different Shade Selection Methods: A Systematic Review and Meta-Analysis. Materials.

[7] Abdelraouf R.M., Habib N.A. (2016). Color matching and blending effect of universal shade bulk fill resin composite in resin composite models and natural teeth. BioMed Res. Int..

[8] Lee Y.K. (2007). Influence of scattering/absorption characteristics on the color of resin composites. Dent. Mater..

[9] Lee Y.K., Lu H., Powers J.M. (2005). Measurement of opalescent property of resin composites. Dent. Mater..

[10] Oivanen M., Keulemans F., Garoushi S., Vallittu P.K., Lassila L. (2021). The effect of refractive index of fillers and polymer matrix on translucency and color matching of dental resin composite. Biomater. Investig. Dent..

[11] Sakaguchi R.L., Douglas W.H., Peters M.C. (1992). Curing light performance and polymerization of composite restorative materials. J. Dent..

[12] Pilo R., Oelgiesser D., Cardash H.S. (1999). A survey of output intensity and potential for depth of cure among light-curing units in clinical use. J. Dent..

[13] Flury S., Peutzfeldt A., Lussi A. (2014). Influence of increment thickness on microhardness and dentin bond strength of bulk fill resin composites. Dent. Mater..

[14] Lucena C., Ruiz-López J., Pulgar R., Della Bona A., Pérez M.M. (2021). Optical behavior of one-shaded resin-based composites. Dent. Mater..

[15] Lowe R.A. (2019). OMNICHROMA: One Composite That Covers All Shades for an Anterior Tooth. Compend. Contin. Educ. Dent..

[16] Graf N., Ilie N. (2022). Long-term mechanical stability and light transmission characteristics of one shade resin-based composites. J. Dent..

[17] Pereira Sanchez N., Powers J.M., Paravina R.D. (2019). Instrumental and visual evaluation of the color adjustment potential of resin composites. J. Esthet. Restor. Dent..

[18] Altınışık H., Özyurt E. (2023). Instrumental and visual evaluation of the color adjustment potential of different single-shade resin composites to human teeth of various shades. Clin. Oral Investig..

[19] Chen F., Toida Y., Islam R., Alam A., Chowdhury A.F.M.A., Yamauti M., Sano H. (2021). Evaluation of shade matching of a novel supra-nano filled esthetic resin composite employing structural color using simplified simulated clinical cavities. J. Esthet. Restor. Dent..

[20] Chen F., Wu D., Islam R., Toida Y., Kawamoto C., Yamauti M., Sano H. (2022). Evaluation of Color and Spectral Behavior of a Novel Flowable Resin Composite after Water Aging: An In Vitro Study. Materials.

[21] Kang S., Ryu S.Y., Kim K.M., Park S.H. (2023). Effect of thickness on the translucency of resin-based composites and glass-ceramics. Dent. Mater. J..

[22] Tsubone M., Nakajima M., Hosaka K., Foxton R.M., Tagami J. (2012). Color shifting at the border of resin composite restorations in human tooth cavity. Dent. Mater..

[23] Medeiros J.A., Pecho O.E., Pérez M.M., Carrillo-Pérez F., Herrera L.J., Della Bona A. (2021). Influence of background color on color perception in dentistry. J. Dent..

[24] Witt K. Excel Spreadsheet Implementation of the CIEDE2000 Color Difference Formula (Including Test Data). http://www.rit.edu/cos/colorscience/rc_usefuldata.php.

[25] Del Mar Perez M., Ghinea R., Herrera L.J., Ionescu A.M., Pomares H., Pulgar R., Paravina R.D. (2011). Dental ceramics: A CIEDE2000 acceptability thresholds for lightness, chroma, and hue differences. J. Dent..

[26] Gómez-Polo C., Portillo Muñoz M., Lorenzo Luengo M.C., Vicente P., Galindo P., Martín Casado A.M. (2016). Comparison of the CIELab and CIEDE2000 color difference formulas. J. Prosthet. Dent..

[27] Paravina R.D., Ghinea R., Herrera L.J., Bona A.D., Igiel C., Linninger M., Sakai M., Takahashi H., Tashkandi E., Perez Mdel M. (2015). Color difference thresholds in dentistry. J. Esthet. Restor. Dent..

[28] Paravina R.D., Pérez M.M., Ghinea R. (2019). Acceptability and perceptibility thresholds in dentistry: A comprehensive review of clinical and research applications. J. Esthet. Restor. Dent..

[29] Khashayar G., Bain P.A., Salari S., Dozic A., Kleverlaan C.J., Feilzer A.J. (2014). Perceptibility and acceptability thresholds for color differences in dentistry. J. Dent..

[30] Xu B.T., Zhang B., Kang Y., Wang Y.N., Li Q. (2012). Applicability of CIELAB/CIEDE2000 formula in visual color assessments of metal-ceramic restorations. J. Dent..

[31] Paravina R.D., Majkic G., Imai F.H., Powers J.M. (2007). Optimization of tooth color and shade guide design. J. Prosthodont..

[32] Horie K., Nakajima M., Hosaka K., Kainose K., Tanaka A., Foxton R.M., Tagami J. (2012). Influences of composite-composite join on light transmission characteristics of layered resin composites. Dent. Mater..

[33] Sarafianou A., Kamposiora P., Papavasiliou G., Goula H. (2012). Matching repeatability and interdevice agreement of 2 intraoral spectrophotometers. J. Prosthet. Dent..

[34] Igiel C., Lehmann K.M., Ghinea R., Weyhrauch M., Hangx Y., Scheller H., Paravina R.D. (2017). Reliability of visual and instrumental color matching. J. Esthet. Restor. Dent..

[35] Sampaio C.S., Atria P.J., Hirata R., Jorquera G. (2019). Variability of color matching with different digital photography techniques and a gray reference card. J. Prosthet. Dent..

[36] Mohammadi A., Bakhtiari Z., Mighani F., Bakhtiari F. (2021). Validity and reliability of tooth color selection by smartphone photography and software applications. J. Indian Prosthodont. Soc..

[37] Chu S.J., Trushkowsky R.D., Paravina R.D. (2010). Dental color matching instruments and systems. Review of clinical and research aspects. J. Dent..

[38] Arimoto A., Nakajima M., Hosaka K., Nishimura K., Ikeda M., Foxton R.M., Tagami J. (2010). Translucency, opalescence and light transmission characteristics of light-cured resin composites. Dent. Mater..

[39] Ghinea R., Pérez M.M., Herrera L.J., Rivas M.J., Yebra A., Paravina R.D. (2010). Color difference thresholds in dental ceramics. J. Dent..

[40] Technical Report—OMNICHROMA FLOW. https://omnichromaflow.com/wp-content/uploads/2021/02/OMNICHROMA-FLOW-Tech-Report.pdf.

[41] Arai Y., Kurokawa H., Takamizawa T., Tsujimoto A., Saegusa M., Yokoyama M., Miyazaki M. (2021). Evaluation of structural coloration of experimental flowable resin composites. J. Esthet. Restor. Dent..

[42] Aida A., Nakajima M., Seki N., Kano Y., Foxton R.M., Tagami J. (2016). Effect of enamel margin configuration on color change of resin composite restoration. Dent. Mater. J..

[43] Leyva Del Rio D., Johnston W.M. (2022). Optical characteristics of experimental dental composite resin materials. J. Dent..

[44] Azhar G., Haas K., Wood D.J., van Noort R., Moharamzadeh K. (2019). The Effects of Colored Pigments on the Translucency of Experimental Dental Resin Composites. Eur. J. Prosthodont. Restor. Dent..

[45] Nakajima M., Arimoto A., Prasansuttiporn T., Thanatvarakorn O., Foxton R.M., Tagami J. (2012). Light transmission characteristics of dentine and resin composites with different thickness. J. Dent..

[46] Son S.A., Park J.K., Seo D.G., Ko C.C., Kwon Y.H. (2017). How do light attenuation and filler content affect the microhardness polymerization shrinkage and translucency of bulk-fill composites?. Clin. Oral Investig..

